# Collective animal navigation and migratory culture: from theoretical models to empirical evidence

**DOI:** 10.1098/rstb.2017.0009

**Published:** 2018-03-26

**Authors:** Andrew M. Berdahl, Albert B. Kao, Andrea Flack, Peter A. H. Westley, Edward A. Codling, Iain D. Couzin, Anthony I. Dell, Dora Biro

**Affiliations:** 1 Santa Fe Institute, Santa Fe, NM 87501, USA; 2 School of Aquatic and Fishery Sciences, University of Washington, Seattle, WA 98195, USA; 3 Department of Organismic and Evolutionary Biology, Harvard University, Cambridge, MA 02138, USA; 4 Department of Migration and Immuno-Ecology, Max Planck Institute for Ornithology, 78315 Radolfzell, Germany; 5 Department of Biology, University of Konstanz, 78457 Konstanz, Germany; 6 Department of Fisheries, University of Alaska Fairbanks, Fairbanks, AK 99775, USA; 7 Department of Mathematical Sciences, University of Essex, Colchester, CO4 3SQ, UK; 8 Department of Collective Behaviour, Max Planck Institute for Ornithology, Konstanz, Germany; 9 Chair of Biodiversity and Collective Behaviour, University of Konstanz, 78457 Konstanz, Germany; 10 National Great Rivers Research and Education Center, Alton, IL 62024, USA; 11 Department of Biology, Washington University in St Louis, St Louis, MO 63130, USA; 12 Department of Zoology, University of Oxford, Oxford OX1 3PS, UK

**Keywords:** migration, animal culture, many wrongs, leadership, collective learning, emergent sensing

## Abstract

Animals often travel in groups, and their navigational decisions can be influenced by social interactions. Both theory and empirical observations suggest that such collective navigation can result in individuals improving their ability to find their way and could be one of the key benefits of sociality for these species. Here, we provide an overview of the potential mechanisms underlying collective navigation, review the known, and supposed, empirical evidence for such behaviour and highlight interesting directions for future research. We further explore how both social and collective learning during group navigation could lead to the accumulation of knowledge at the population level, resulting in the emergence of migratory culture.

This article is part of the theme issue ‘Collective movement ecology’.

## 1. Introduction

Animal movement is a fundamental driver of ecological and evolutionary processes. Movement, and specifically migrations, couple disparate populations and ecosystems by transporting individuals, nutrients, pathogens and genes [1,2]. For individuals, migrations facilitate access to spatially and temporally varying resources; however, there are significant costs and challenges associated with migration [3]. Perhaps the most serious challenge is navigation—animals must find their way through often complex environments along migration routes that can span tens of thousands of kilometres and take many months (sometimes generations) to traverse. To successfully complete these migrations, animals employ a diverse range of sensory modalities and can respond to an impressive array of cues, including magnetic fields, light polarization, landmarks, odours and celestial bodies [4]. While in some contexts the preferred navigation route is genetically encoded and instinctive, for others this must be discovered or learned from others.

**Figure 1.**
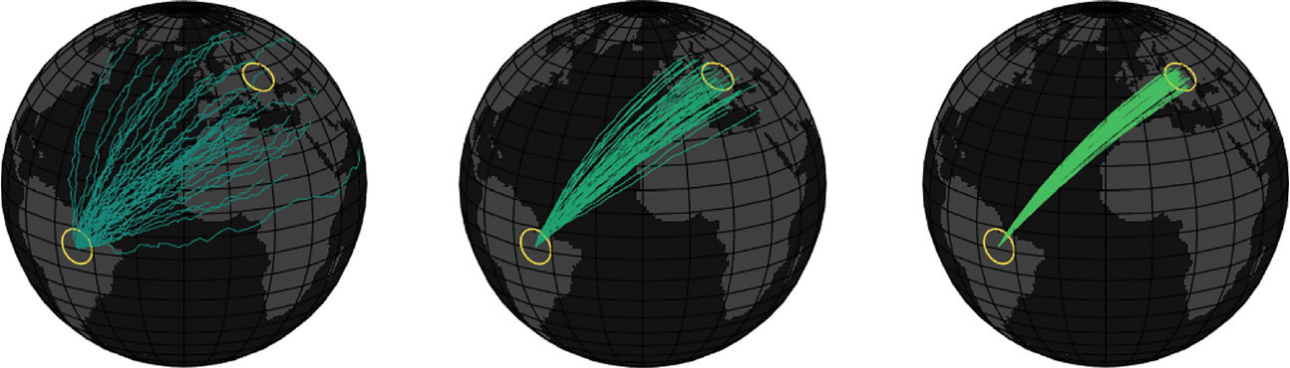
Illustration of the potential benefit of collective navigation. In this hypothetical example, migrants seek to travel from South America to Europe, with each line denoting a particular group of migrants. On average the navigation accuracy improves from left to right, which could be due to an increase in the size of the group, increase in the fraction of leaders in the group, or learning by individuals. See box 1 for details of collective navigation mechanisms. In reality, the ‘best’ route may not be the straightest path, as navigational efficiency will be a function of several considerations, including resource distribution, perceived safety and cumulative hydro/aerodynamic efficiency.

Although the mechanisms of animal navigation have fascinated researchers for decades, focus has primarily been at the level of the individual [4]. However, many migratory species are known to move in large groups [5] and social interactions can alter migratory movement decisions [6,7]. How individual navigational ability is affected by social interactions, and what unique orientational capacities can emerge at the collective level, has been far less studied, although a growing body of theoretical and empirical results supports the hypothesis that social interactions during collective navigation can lead to improved navigational ability (figure 1). We define *collective navigation* as the outcome of navigating within a social context. These outcomes can be beneficial, neutral or detrimental, although we note that for the most part we, and the field in general, focus particularly on positive outcomes.

Here we review the growing literature on collective navigation in order to: (i) provide an overview of the theoretical mechanisms by which social interactions can facilitate navigational benefits; (ii) synthesize empirical support for these mechanisms across several taxa, both in controlled experiments and in observations from the field; (iii) explore how social and collective learning may allow for the accumulation of information at the population level, thus leading to the emergence of animal culture in a migratory context and (iv) highlight potentially fruitful directions to further the study of collective animal navigation, especially with the use of new technologies.

We describe five broad mechanisms for collective navigation: many wrongs, emergent sensing, leadership, social learning and collective learning (box 1). The first three describe different ways in which social interactions may lead to improved navigation during a single navigational bout. Social and collective learning (see box 1d,e for distinction between these two) describe how information can propagate through a population or across generations, and how new information can emerge through social interactions. While previous reviews tend to focus on specific mechanisms (e.g. many wrongs [8], leadership [9], social learning [10]), here we focus on these mechanisms in the context of navigation, and highlight differences between, and interactions across, the various mechanisms. Hence, we show that the five mechanisms are not mutually exclusive, and collective navigation can be the result of a complex and dynamic set of processes spanning multiple spatial and temporal scales.

Box 1. **Mechanisms leading to improved accuracy during collective navigation.**

**Figure 2.**
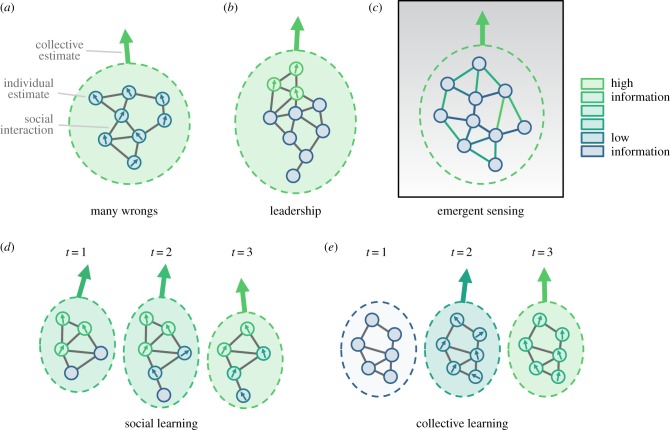
Mechanisms leading to improved accuracy during collective navigation. (*a*) Many wrongs: noisy estimates from many individuals are averaged to produce a more accurate collective estimate. (*b*) Leadership: a subset of informed individuals guides naive individuals. (*c*) Emergent sensing: comparisons of individual measurements of the environment via social interactions allows a group to detect gradients. Here information is present in the interactions (links) rather than the individuals themselves. (*d*) Social learning: navigational information passes from informed individuals to naive individuals over time. (*e*) Collective learning: new navigational information is generated over time through social interactions.

(a) ***Many wrongs*** is the mechanism by which a group of animals, each with a noisy estimate of the ‘correct’ navigation direction, can improve their accuracy by pooling individual estimates. At its core, it is deeply related to the law of large numbers. As long as the errors of individual estimates are not perfectly correlated with each other, and are distributed in an unbiased manner around the true value, then a simple averaging across estimates can increasingly dampen noise and home in on the true value (figure 2*a*). Known social interaction rules have been shown to effectively average across preferences. This mechanism can operate on either continuous (such as direction of motion) or discrete (such as distinct paths or river branches) variables. In the latter case, majority (or plurality) rule serves an analogous function to simple averaging. For a group composed of individuals with differing accuracies, many wrongs may still improve accuracy, although accuracy would be maximized by a weighted average.
(b) ***Leadership*** results when informed individuals, which may form a small minority of the group, successfully guide naive individuals towards favourable environments. Smaller groups may allow for individuals to recognize leaders and preferentially follow them, while in large groups, leaders are likely to be anonymous. Nonetheless, social influence can lead to successful leadership, with a surprisingly small number of leaders necessary for accurate navigation (figure 2*b*). Naive individuals can even help ensure democratic decision-making, potentially aiding in a many-wrongs improvement of accuracy. Who is a leader can depend on the specific context, so that over the course of a migration, leadership may be distributed among many members of the group.
(c) ***Emergent sensing*** occurs when a group can navigate collectively even when no individual has the ability to assess the correct direction of motion. If an individual, for example, can make only scalar measurements of an environmental cue and has no memory, then it has no knowledge of the gradient of the cue. But a group can, collectively, measure and follow a gradient if the measurements made by multiple individuals can be compared. The group would then function as a distributed sensor network. Although many animals that navigate together cannot directly communicate and compare measurements with each other, context-dependent behaviour (where some aspect of behaviour is tied to the value of the measurement) can effectively facilitate such comparisons, even if no individual is aware of them (figure 2*c*).
(d) ***Social learning*** allows knowledge possessed by informed individuals to percolate through the group and across generations. If naive individuals are led along a particular path by more knowledgeable group members, those individuals may learn about cues associated with that path, therefore becoming part of the informed subset themselves over time. Similarly, individuals with similar ages, or levels of experience, may have differing knowledge of specific routes or cues and this information may be homogenized via learning during group travel. In both contexts, the learning is unidirectional—individuals gain personal information by following others who already have that information. For navigational tasks where there is no genetically encoded preferred direction, social learning can be the primary mechanism by which navigational information persists over generations. Innovations to routes (e.g. novel shortcuts, detours) originate with leaders/demonstrators at the individual level, and can be passed on to followers/observers.
(e) ***Collective learning*** is the emergence and retention of new knowledge resulting from the dynamics of social interactions. It differs from social learning in that route innovations are generated from the interaction of multiple individuals. For example, a group can improve the route that it takes through the many wrongs mechanism, and this new route can then be learned by individuals in the group. Alternatively, naive individuals may inject random noise (stochastic factors such as sensory, or movement, errors) into a travelled route, and improved routes could be haphazardly discovered and subsequently learned—although this may require the group to also have the capacity to filter out ‘bad’ innovations. Both collective and social learning may lead to gradual improvements, or ‘ratcheting’, of the efficiency of the learned route over time.

These mechanisms may also apply to many other navigational tasks in addition to migrations. For example, many animals navigate in order to discover new food sources, move up and down the water column, or locate new shelters. Because animals use environmental information to reach specific targets in these and other tasks, collective navigation mechanisms could play a role for group-living animals in improving their performance. Furthermore, while the majority of the direct empirical evidence for collective navigational mechanisms uses birds or fish as study organisms, there are many other taxa, including ungulates, cetaceans and insects, which navigate through their environment while travelling in groups. Where relevant, we allude to some of these less well studied taxa as potential directions for future research.

## 2. Theoretical models and mechanisms

The idea that the effectiveness of a collective decision-making process covaries with group size dates back several centuries, initially focusing on decision-making in humans. One classic example, from the late eighteenth century, is Condorcet's jury theorem, which posits that when individuals must choose between two discrete options (e.g. the guilt or innocence of a defendant), and each jurist has a greater than 50% chance of choosing the correct option, then the accuracy of decisions will tend to improve as the size of the group increases [11]. Later work, including that of Galton [12], extended this idea from discrete to continuous estimates, suggesting that the average of many independent estimates will tend to approach the ‘true’ value with increasing accuracy as group size increases—a phenomenon now known as the ‘wisdom of crowds’.

It was only much later that these ideas were adapted to non-human animal groups when, in the 1960s, researchers studying birds [13–15] and fish [16] independently suggested that these animals could improve their navigational performance by grouping. For example, Larkin & Walton [16] supposed that each fish within a school makes an independent estimate of the best migratory direction, and by travelling together they would tend to move in the average preferred direction of all individuals. In such a scenario, assuming there is no cost to aggregating information, navigational error should decrease as the inverse of the square root of the number of animals in the group, analogous to how the standard error shrinks as the sample size increases in statistical analyses due to the law of large numbers. Similarly, Condorcet's theorem could apply in animal groups when animals must make binary or other discrete choices, such as fish ascending a river network [17] or bees selecting a new nest site [18], such that decision accuracy improves with group size in these scenarios [19]. Now known as the ‘many wrongs principle’, the general idea that social interactions dampen individual errors is thought to be a major outcome of collective navigation ([8]; box 1a).

While these relatively simple mathematical arguments provide an intuitive conceptual basis for how individuals in groups could improve their navigational accuracy, they largely ignore the complexity of the behaviour of real organisms. In most animal groups, there is no entity to collate ‘opinions’ and explicitly compute the average of all individual estimates, as each individual can observe only near neighbours. Furthermore, individuals may not be equally informed about the best direction of travel, there may be complex interactions between genetically determined and learned preferences, or group-wide biases in estimates. Because of this, it is not obvious whether navigational accuracy in animal groups would scale as these simple models predict, or whether there are limits to the real-world ability of organisms to benefit from collective navigation. More detailed models are necessary to shed greater light on the mechanisms underlying collective navigation in animals.

Agent-based models, where the motion of each individual is modelled explicitly in space and time [20,21], were developed in order to bridge the gap between abstract mathematical models and the behaviour of real animal groups. These models can describe how the motion of an individual is determined by its own navigational preferences, physical abilities, sensory information and response to near neighbours. The social interaction rules are often governed by ‘zones’ of interactions, such that the response to a neighbour depends on the distance between the neighbour and the focal individual [22–25]. More recently, empirical data have driven the development of alternative models, where, for example, individuals respond to a fixed number of near neighbours irrespective of their distance [26], where social influence decays continuously as a function of distance [7] or where interactions are modulated by considerations of the animals' sensory capacities and limitations [27,28]. Agent-based models are particularly useful because ‘experiments’ can be performed *in silico* even when the underlying equations are not mathematically tractable. Furthermore, experiments can be performed digitally to address questions that may be difficult or impossible to do with real animals in the laboratory or the field. For example, different parameters of the model (such as sensing ability, social interaction network or the structure of noise) can be varied systematically, and their effect on collective navigation measured. In addition, such models allow an exploration of how collective behaviour may change over evolutionary timescales [25,29]. The results of such virtual experiments can serve as testable predictions regarding which behavioural parameters are likely to be important for real animals, which can lead to more targeted experiments.

The simplest agent-based models of collective navigation assume that all individuals in the group are identical—they follow the same interaction rules and have the same level of navigational information or error, thus approximating the conditions that the many wrongs principle typically assumes. Such simulations have demonstrated that many-wrongs averaging can readily arise from local social interactions if individuals balance their own preference with the direction of motion of their neighbours [30,31]. Specifically, collective navigational performance is maximized when personal preference is given a low weight [32], if individuals exhibit some inertia in their movements (which serves to average an individual's noisy compass estimates over time) [33], or if the underlying social structure is evenly distributed, rather than dominated by a few individuals [34,35].

For many other contexts, the distribution of directional preferences may be multimodal rather than unimodal. For example, different individuals in a group may have different preferred routes to the same location, and at small spatial scales, individuals can exhibit distinct preferred headings. In other cases, individuals may prefer altogether separate locations, such as when individuals in a breeding population choose from multiple overwintering grounds (i.e. weak migratory connectivity [36]). In such cases, there will be a natural continuum between unimodal and multimodal distributions of preferences depending on the distance individuals are from the final location. Specifically, when locations are very far away, all individuals prefer to move roughly in the same direction (unimodal), but as the group approaches the locations preferences will begin to diverge (become multimodal). In such scenarios, simply taking the average of the preferred directions can be detrimental (there may well be no suitable habitat at the midpoint between preferred locations). Agent-based models that incorporate this diversity of preferences have demonstrated that, despite these challenges, groups are consistently able to reach consensus for one particular location. One robust result of both models and empirical data is that animal groups average when the discrepancy between preferred headings is small, but when the discrepancy is sufficiently large, the group spontaneously selects one of the possible headings [24,37,38], typically the one preferred by the greatest number of individuals [24,37,38] or the most strongly opinionated individuals [39,40].

Another realistic extension of these agent-based models is to include two classes of individuals, informed and naive, where successful navigation requires leadership by the informed class (box 1b). In real animal groups, this can occur when the desired navigation direction is not genetically encoded and must be learned: the naive individuals may be juveniles that lack experience of the route, or members of fission–fusion groups that are less knowledgeable about the local geography or other informative cues. One question that arises from these mixed groups is whether, and how, relevant information about which way to go can successfully percolate from a minority of leaders to the entire group. Effective leadership would not be explained by many wrongs, which would predict poor navigational ability in such scenarios, as it describes the averaging of estimates across the entire group. This challenge is compounded if information about who is informed cannot be directly signalled, and leadership must arise despite this anonymity. Models in which a group is composed of an informed subclass and an uninformed subclass show that surprisingly few informed individuals are necessary to effectively lead a group [24,41,42], with a relatively sharp transition from ineffective to effective leadership. Models suggest that leadership can be enhanced if the informed subclass moves more quickly than the naive majority [43] in order to increase their contact rate or to signal information, although this is not a requirement for effective leadership [24]. Further studies have shown that naive individuals can even improve collective navigation, because they contribute error that can actually stabilize consensus decision-making and increase the speed and sensitivity of consensus [44,45].

Knowledge heterogeneity may be an outcome of evolution, rather than simply a consequence of age structure or mixing. Evolutionary simulations, in which gathering information is costly (as it necessitates, for example, developing enhanced sensory capabilities or diverting more attention to information gathering) suggest that frequency-dependent selection drives the evolution of leaders (those who predominantly rely on environmental cues) and followers (those who predominantly rely on social cues) [29,46]. This may even occur when individuals are very sparsely distributed in space, and thus rarely interact, demonstrating that individuals can benefit from ‘collective’ navigation even if they do not appear to be grouping at all [29].

Differential levels of knowledge also provide opportunities for naive individuals to learn migratory routes and other relevant information socially for use in future journeys. Such unidirectional copying behaviour is typically referred to as social learning [47] (box 1d). Hamilton [48] and others proposed the intuitive idea that young migrants could learn migration routes when travelling with more experienced individuals by being exposed to cues associated with that route. Social learning may also occur between individuals of the same age class. For example, in fission–fusion populations, there may be local heterogeneity in knowledge about the environment due to the mixing of individuals among groups [49]. In such scenarios, animals can gain information about relevant geographical features or landmarks by following better informed, transient, group members. While the role of social learning in collective navigation has received substantial empirical support (which we discuss in a later section), there are fewer theoretical models. However, the models that do consider the transmission of information across generations suggest that it could lead to collective memory in a population, allowing for migration routes and destinations to be culturally established and maintained [42,50,51].

In addition to social learning, whereby information is passed from one individual to another (or several others), social interactions can also lead to collective learning, where new information emerges *de novo* as a result of social interactions (box 1e). For example, a group can jointly discover an improved route, through many wrongs or randomly by noise injected from social interactions, which can then be learned by the group members. Kao *et al.* [52] demonstrated theoretically that the collective context within which decisions are made can substantially alter what individuals learn about their environment, enabling them to maximize collective accuracy without the need for special social cognitive abilities.

By altering how individuals experience the world, social interactions can affect what aspects of the environment are learned and can contribute to new knowledge within the group that improves navigation. Such learning can lead to the accumulation of increasingly better navigational solutions over time, in a process analogous to cumulative cultural evolution [53]. We return to both social and collective learning in a later section, and provide more explicit suggestions for the key aspects that differentiate them, as well as for the consequences that these differences have for what form migratory cultures take.

While the above models largely presumed a preferred absolute travel direction or target, in many contexts animals navigate by following local cues. Additionally, animals may perform local search to find winds or currents that are favourable for their migration route [54]. In these scenarios, successful navigation can require detecting and climbing environmental gradients, such light, odour, temperature or current [4]. In theory, a group could act as a spatially distributed sensory array spanning weak environmental gradients and amplifying weak signals [55–58]. In such a scenario, the many wrongs effect (box 1a) could help a group climb a noisy environmental gradient if each individual makes an independent assessment of the direction of the gradient [30,31].

However, effective climbing of gradients can also occur collectively even when individuals themselves are unable to detect gradients. Known as emergent sensing, social interactions facilitate comparisons across scalar measurements made by individuals, leading to a collective computation of the environmental gradient [56–58] (box 1c). For example, by altering individual-level behaviour (e.g. social interactions [56] or swim speed [58]) in response to local scalar values of the environment, movement up a gradient can emerge at the group level. Hein *et al.* [25] used simulations to demonstrate such group-level traits are an evolutionarily stable outcome, readily arising from selection operating on the behaviour of selfish individual agents rather than explicitly on group-level properties. In contrast to the many wrongs effect, which has a known upper bound to accuracy, the limits of emergent group sensing are not well understood. The space of such context-dependent behavioural rules is potentially very large and much remains to be explored, both theoretically and empirically. Because current techniques to infer social interaction rules from data typically average over time and individuals, they potentially miss such context-dependent behaviours that may be highly relevant to navigation.

## 3. Signatures in the wild

The theoretical and modelling work on collective navigation make a number of broad predictions about the movement of animals in the wild. A few prominent examples include: (i) larger groups should, on average, navigate more accurately than smaller groups; (ii) a small proportion of informed leaders should be able to effectively lead a large group; (iii) larger groups should better sense and respond to their environment and (iv) individuals should be able to learn to improve their own navigational knowledge or ability by socially facilitated exposure to relevant environmental cues. One avenue by which to study collective navigation empirically is to compare these theoretical predictions to observational data from the wild. Observations that appear to agree with these theoretical predictions would not conclusively demonstrate collective navigation in these species but would highlight potentially relevant species for further experimental study. In this section, we summarize observations of real animals—primarily in migratory species—that are consistent with predicted outcomes of collective navigation (also see table 1).

**Table 1.** Summary of selected collective navigation studies categorized by the primary mechanism and type of evidence. In entries marked with an *, the exact mechanism is not clear.

EVIDENCE			
	models	signatures from the wild	experiments
MECHANISMS			
many wrongs	[15,16,30–32,34,35]	common scoter [13]	homing pigeons [37,59,61,63,64]
		white storks [60]	king penguins [65]
		skylarks [62]	larval damselfish [66]
		salmon [17]*	mosquitofish [67]*
			humans [68]*
leadership	[24,29,43,46]	whooping cranes [69]	sticklebacks [70]
		white storks [71]	homing pigeons [37,73]
		Atlantic herring [72,74]	white storks [75,77]
		short-toed eagles [76]	golden shiners [44,79,82]*
		orcas [78]	guppies [83]
		bottlenose dolphins [80]	honeybees [84]
		African elephants [81]	
emergent sensing	[25,56–58]	wildebeest [85]*	golden shiners [58]
		salmon [86]*	
		white storks [60,71]*	
social learning	[42,50,51]	whooping cranes [69]	white storks [75,88]
		Atlantic herring [87]	starlings [90]
		brent geese [89]	French grunts [91]
			honeybees [92]
			Temnothorax ants [93]
collective learning	[52]	Atlantic herring [74]*	homing pigeons [94,95]
			bluehead wrasse [96]*

The earliest observational studies focused on the many wrongs principle (box 1a) in migrating birds. Consistent with the predictions of this principle, directional accuracy appears to increase with group size for fowl [13], white storks [60] and skylarks [62], although the latter study [62] is limited due to a small range of group sizes. More recently, experimental studies using GPS-tracked individuals have yielded more rigorous support for many wrongs [37,64] (see next section for details).

Migrations that rely on local cues for effective navigation provide support for the theory of emergent sensing (box 1c). Congruent with predictions of emergent sensing, storks in flocks are better than individuals at locating thermal updrafts along their migration route, which the birds use to gain altitude more efficiently [60]. Further, wildebeest move towards new food resources that are ostensibly beyond their personal sensory range [85], although an alternate explanation is that rain clouds or lightning flashes may be visible over large distances and provide meaningful information to individuals.

We see evidence of leadership (box 1b) in the wild, both within and between generations. Predictions that distinct leader and follower behavioural types exist within a generation [29,46] are supported by recent empirical evidence from a flock of wild white storks. Nagy *et al*. [71] found that during their first migration a relatively small subset of individuals act as leaders both within, and between, thermals. Leaders needed to constantly adjust their flight paths to locate regions of maximal lift within the complex physical environment of thermals, whereas followers, by exploiting social information, exhibited more efficient paths. However, these followers left thermals earlier, and at lower altitudes, resulting in them exhibiting considerably more flapping flight as they moved between thermals. In support of the idea of inter-generational leadership, Mueller *et al*. [69] found that navigational accuracy increased with the age (a proxy for experience) of the oldest bird in a group, and not as a function of group size (as many wrongs would predict) in a population of reintroduced whooping cranes (*Grus americana*). Thus, in this system, younger birds benefit from travelling with older, more experienced, birds. Similarly, experienced older and/or more dominant individuals show disproportionate leadership in group-living mammals with stable social structures, such as orcas (*Orcinus orca*) [78], elephants (*Loxodonta* sp.) [81] and wolves (*Canis lupus*) [97]. Further, in Atlantic herring (*Clupea harengus*) the establishment of new migratory destinations coincides with peaks in the ratio of first-time spawners to repeat spawners [72,74]. This suggests that the large influx of naive migrants swamps the ability of the older, informed, fish to lead—though an alternative (or additional) hypothesis is that the naive individuals have a greater affinity to (collectively) track environmental gradients than do experienced individuals [74].

Navigating in groups with inter-generational leadership can also lead to social learning (box 1d). In fact, Mueller *et al.*'s [69] original generation of cranes succeeded to learn a migration route ‘socially’ from an ultralight aircraft. Although subsequent generations learning from older individuals was not directly tested, the phenomenon could be reasonably inferred from the data. Similarly, for Atlantic herring, genetic or environmental factors do not explain well this species' annual return to specific sites to feed and breed, leaving social learning, where young individuals school with and learn from older and more experienced individuals, as the most likely explanation [87,98]. Results from studies of light-bellied brent geese (*Branta bernicla hrota*) show that most offspring chose staging and wintering sites in adulthood that were identical or very near to those of their parents, suggesting an important role of social learning of migratory routes, as limited genetic differences between migrants from different routes was observed [89].

Often the specific mechanism underlying collective navigation is not apparent, but consistent patterns of generally increased navigational ability with increasing density reveal a potential signature of this process. For example, Keefer *et al.* [86] performed a statistical analysis of factors influencing the rate of salmon movement in various river conditions and showed that adult salmon are able to pass more quickly through artificial barriers—hydroelectric dams—at high densities. Berdahl *et al.* [17] performed a meta-analysis of the relationship between homing rates and the number (density) of migratory fish in Pacific and Atlantic salmon and found a consistent trend in which years of greater abundance of fish were associated with more accurate navigation to natal streams. These results could be the net effect of several mechanisms acting in parallel or in series: salmon may benefit from many wrongs when crossing the high seas (continuous estimates), consensus decision-making when choosing between two river tributaries (discrete options) and emergent sensing when locating the odor plume of a river estuary or entrance of a fish ladder.

An additional, albeit even less direct, line of evidence for animals benefitting from collective navigation may come from the interplay between population and migratory dynamics. Theory suggests that populations employing social navigation strategies may be prone to collapse and cease migration at low population size [50,99]. This predicted collapse is due to an Allee effect, whereby positive feedback between reduced population size and reduced benefits from collective navigation (regardless of mechanism) leads to further reductions in the population size. Indeed, sudden population collapse has been observed in many group migrating species [100]. Further, migratory distance in wildebeest may be linked to population size [101,102] and in the case of caribou, migrations have stopped altogether when population sizes became low, only to recover when the number of animals increased [103].

## 4. Experimental evidence of collective navigation

While field observations are typically only correlative and may be subject to a confirmation bias, controlled experiments can establish a causal link between one or more collective navigation mechanisms and the resulting performance of the group. However, even in controlled experiments it can still be often difficult to distinguish between various mechanisms [104]. Here, we review several prominent examples of experiments that have demonstrated collective navigation, where the benefits range from transient improvements to longer lasting effects of socially facilitated learning (also see table 1).

The spatial scale of laboratory-based experiments is typically limited and, as such, these are often only amenable to the study of smaller-scale challenges. However, many navigational tasks faced by animals operate on similar scales, and even many long-distance movements are guided by a series of local interactions with the environment. Laboratory experiments can therefore shed light on the mechanisms governing collective navigation in nature. For example, Ward *et al.* [67] showed that larger groups of mosquitofish (*Gambusia holbrooki*) make faster and more accurate binary decisions than do smaller groups. While the challenge in that particular experiment was to avoid predation, the general result may be applicable to migratory groups encountering binary choices, such as anadromous fish homing to a particular branch of a river network [17]. Emergent sensing can also be studied and revealed in the laboratory. Taking advantage of the innate preference of golden shiners to low light environments, Berdahl *et al.* [58] demonstrated that the ability to climb environmental gradients increases with group size. The researchers found that when individual fish modulate their swimming speed in response to the local brightness level, taxis was induced at the group level, even though individuals had little ability to sense the gradient themselves. Laboratory experiments have also shown that collective navigation can emerge from the pooling of differential information across individuals. This pooling can occur for a single decision, for example, if subgroups are knowledgeable about different informational dimensions (cues) and reach a consensus about an option that contains both cues [82], or across a series of decisions, for example, from the dynamic allocation of leaders depending on which subgroup has the relevant information for that particular decision [70]. Simple mechanisms like these may underlie a variety of, as yet poorly understood, situations in which groups navigate in response to local cues.

Experiments can also be performed outside of the laboratory. One fruitful method is to take advantage of the natural homing behaviour in some animals. In such cases, group size and composition can be easily manipulated and both the start and endpoints can be controlled, while taking place under naturalistic conditions. Early experiments using homing pigeons (*Columba livia*) showed conflicting results—some demonstrated a benefit of flocking on homing performance [63] while others did not [59,61]. However, these early studies assessed navigational performance only by examining the directional orientation of the birds at the release site (i.e. ‘vanishing bearings’) and the total time birds took to reach home, rather than the structure of complete trajectories. As such, they only provide rather crude measures of navigational performance. Such limitations have been overcome with the advent of miniature GPS technology that now provides high-resolution tracks of entire journeys, allowing for more detailed analyses of the selected routes. Using this technology, researchers have shown that pigeons in flocks tend to have straighter routes than when flying alone, suggesting that the group's route comprises an averaged direction that is more accurate than individual estimates [37,64]—a form of many wrongs in operation. Similar homing experiments have been performed in other non-domesticated species. For example, groups of king penguin chicks (*Aptenodytes patagonicus*) returned to their crèches faster and via more efficient routes after displacement than did solo chicks [65], while larval damselfish *Chromis atripectoralis*, homing to their natal reef, swam straighter and faster in groups than they did when swimming individually [66].

Homing experiments can also test whether a collective improvement can persist beyond the one-off experience of a given flock flight, by influencing individual orientational performance long-term through social or collective learning. In pigeons, naive individuals not only follow more experienced leaders [73] but also socially learn the demonstrated homing routes while doing so, evidenced by their ability to recapitulate these learned routes during subsequent solo flights [95]. However, a single demonstration of a route seems to be insufficient for such transfer to occur [105], with robust learning requiring repeated trips [95]. In addition, naive birds have also been shown to have some influence during paired flights [94,95]. Their presence probably injects noise into the decision-making process, which allows the group to try new routes and thus potentially discover improved navigational solutions. Such improvements can persist to subsequent flights (suggesting collective learning), and may even accumulate over successive flights, even when there is continuous turnover within the group [94] (see also next section).

Displacement experiments during natural migrations are another useful and related technique for studying leadership as well as both collective and social learning. Typically, tagged juveniles or adults are translocated from their normal migration route or habitat, and the subsequent route or variance in route choice provides information about the navigation strategies of individuals. Early studies on both starlings (*Sturnus vulgaris*) and white storks (*Ciconia ciconia*) showed that displaced juveniles followed migratory paths that were common for conspecifics in the area where they had been displaced, indicating that displaced juveniles followed local conspecifics to their wintering grounds [88,90]. Thus, the tendency to follow conspecifics tended to override the innate control of migratory path selection in both the starlings and white storks, a pattern confirmed by Mellone *et al.* [76] in their study of the migration of juvenile short-toed eagles (*Circaetus gallicus*). Furthermore, juvenile storks deprived of their social environment during migration, by being contained until all conspecifics have left the breeding grounds, do not migrate in their usual migratory direction but instead show much larger directional scatter [75,77]. These studies were repeated recently using satellite tracking technology, confirming that naive white storks rely heavily on their social environment when selecting migratory routes [75]. The fact that no evidence is reported in these studies for established migratory routes *changing* through the presence of juveniles suggests that social learning, rather than collective learning, is the principal channel for transmission.

Leadership and social learning are firmly established mechanisms for the propagation of spatial information in eusocial insects. In honeybees (*Apis mellifera*), a surprisingly small subset (approx. 5%) of informed individuals can lead an entire colony to a new nest site [106]. In these swarms, leaders appear to exert influence by repeatedly flying through the swarm in the intended direction faster than the other bees [84]. Information is spread through eusocial insect colonies via various forms of social learning, often with an active ‘demonstrator’. Honeybees use the so-called waggle dances to inform nest-mates about the location of foraging opportunities or new nest sites [92]. Individual ants (specifically, *Temnothorax albipennis*) are even argued to ‘teach’ others about the location of suitable nest sites [93] by leading naive ants to relevant targets through tandem runs [107].

Additional evidence of leadership and social learning comes from laboratory and field studies with fish. In the laboratory, guppies and golden shiners follow experienced individuals to feeding sites [44,79,82,83], with evidence in guppies that the routes persist even once the original leaders are removed [83]. In displacement experiments in the field, such persistence can last for multiple years or even generations. In a classic study, Helfman & Schultz [91] translocated French grunts (*Haemulon flavolineatum*) from their home range to an unfamiliar location in which the resident population exhibited fidelity to particular sites and took specific routes between them. The newly transplanted fish subsequently used the local residents' routes and sites and furthermore continued to use them even after all residents had been removed. As no changes to the residents' routes were reported after the introduction of new fish, the most likely mechanism was leadership followed by social learning. Nonetheless, it is possible that over longer timescales, with the accumulation of many repeated group journeys between sites and a continuous population turnover, input from multiple individuals would combine to gradually shift routes, adding a collective learning element. Importantly, in control experiments, in which all residents were removed prior to conducting a transplant, the transplanted fish did not use the residents' sites and routes, ruling out the possibility that all fish—the transplants in the previous treatment as well as the resident fish—were simply responding to the same environmental cues.

Warner [96] demonstrated similar social transmission in the choice of mating sites by bluehead wrasse (*Thalassoma bifasciatum*). When individuals from six reefs were displaced approximately 2 km away to a new reef location that had been cleared of conspecifics, they developed their own mating sites, which were shown to be a statistically random sample of suitable locations. Importantly, the observation that these new mating sites subsequently remained stable for multiple years is taken to indicate the presence of a persistent ‘culture’ of site preference in bluehead wrasse. This brings us to our next section: the emergence of navigational culture from collective navigational phenomena.

## 5. From collective navigation to the emergence of migratory culture

Across the examples so far discussed, the temporal scale at which individuals are influenced by others varies over many orders of magnitude. On the shortest scale, these influences may be the equivalent of ‘social information use’ [47,108], whereby the movement decisions of individuals—such as their direction, timing or speed—are directly influenced by the presence and movement of fellow group members. However, these effects are transient, influencing the moment-to-moment decisions of individuals but with no longer-term consequences. This is how models of collective motion typically depict interactions—as consecutive timesteps. However, as we have discussed above, when individuals travel along a particular route, whether alone or with (and influenced by) a group, they have the opportunity to memorize cues along the route. These memories may feed back to influence navigational performance when the same task is attempted again subsequently, in effect preserving the knowledge over time, potentially over generations [53]. Such cross-generational persistence through learning, and influenced by the animal's social environment, meets criteria for culture: it can give rise to ‘group-typical behaviour patterns, shared by members of animal communities, that are to some degree reliant on socially learned and transmitted information’ [109]. Therefore, we now turn to the pathways through which the mechanisms of collective navigation we have discussed in this review can lead to the emergence of migratory cultures.

**Figure 3.**
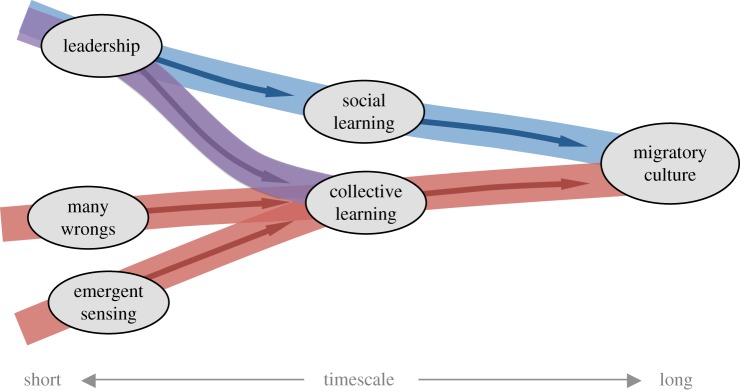
Paths to culture. Schematic summary of different pathways through which mechanisms of collective navigation may lead to navigational culture. Those mechanisms that rely on input from multiple individuals (many wrongs and emergent sensing) create opportunities for culture via collective learning, whereas social learning provides the primary pathway in groups where leadership dominates. See figure 2 and box 1 for more detail on mechanisms.

Figure 3 outlines our two major proposed pathways, with a potential crossover between the two providing a third. First, in systems with despotic leadership, followers have the opportunity for social learning: essentially, they are passive ‘observers’ in the navigational task as they follow knowledgeable (or otherwise appointed) ‘demonstrators’. Observers memorizing routes during these opportunities can lead to the transmission of navigational knowledge, and, if such transmission occurs repeatedly, migratory culture arises. This pathway is likely to operate in cases where, for example, there is little overlap between generations in terms of competence at, or knowledge of, a task, and where leadership is therefore the norm (such as first-time migrants travelling with parents). Second, when groups solve navigational tasks together, and do so through a many wrongs or emergent sensing mechanism, collective learning can replace social learning as the path to cultural transmission. In other words, when solutions to specific navigational problems emerge from pooling individual information-gathering or processing capacities, these collectively derived solutions may be acquired by all of the group's members, and to do so repeatedly over time, giving rise to culture. Third, in cases where leadership is not entirely despotic, but rather graded, input into navigational decisions from followers (albeit weighted less than input from higher-ranked leaders) may provide suitable conditions for collective (rather than purely social) learning. In sum, at the heart of all cultural phenomena are two things: (i) innovations that introduce new behaviours into a population and (ii) non-genetic mechanisms for the transmission of these behaviours. Our three pathways differ in *how the innovations arise* (i.e. through individual or collective intelligence) which in turn influences how they are transmitted (i.e. through social or collective learning, respectively).

Identifying examples of migratory cultures in nature is challenging. It requires multi-generational data that not only tracks the persistence of routes over time, but also confirms that they are maintained via socially mediated transmission. In other words, although it is impossible to fully discount ecological and genetic effects on route choice, these choices should demonstrably be shaped at least partially by the social environment. Furthermore, when route choice shows variation among different populations (or different co-navigating groups) of the same species, especially if moving within the same environment, this can provide important clues to cultural factors being at work. Such data are available from a small number of observational and experimental studies. In the laboratory, transmission-chain designs—a staple of experimental approaches to the study of cultural transmission [110]—have demonstrated the potential for arbitrary travel routes to be passed on via social learning along a succession of leader–follower pairs [83]. In the field, natural transmission chains (such as iterative adult–juvenile joint migrations) are implicated in the maintenance of traditional travel routes [69], while homing and displacement experiments mentioned previously have shown that removing older individuals or an entire resident population can cause an abrupt shift to completely different routes, and even demonstrate that a sufficient number of experienced individuals is necessary for the intra- and inter-generational stability of routes [42,76,91,96]. (In an interesting parallel, such demographic effects feature prominently in the modelling of cultural gain, drift and even loss in human technological evolution [111].)

Individuals in groups do not need to have identical knowledge about the environment, potentially expanding the amount of information available to a group beyond the memory capacity of a single individual. With collective learning, there can be feedback between collective decisions and individual learning (individuals learn about what they experience, and what they experience is affected by the preferences and decisions of others), such that individuals in the same group may actually not learn identical representations of the same environment [52,112]. This can lead to a ‘collective memory’, whereby the environment is represented in the group in a distributed manner. This distributed information can then be accessed, for example, by the dynamic allocation of leaders as different informative cues arise during navigation, as discussed earlier [70,82].

The external environment can also help to reinforce particular routes by serving as a substrate on which memories can be encoded. For example, animals on the move can wear down the vegetation and create clear paths through the landscape. Because following these paths can be less energetically costly than generating one *de novo*, subsequent animals often adopt existing paths, further demarcating them. Olfactory cues left in the environment can also indicate the route taken by others [113]. Stigmergic mechanisms such as these provide a means of social information transfer among individuals separated in time, potentially allowing for extended influence to other conspecific groups or even different species [114,115].

While some routes can be highly entrenched (by persisting relatively unchanged over long time scales), other paths may be further modified and improved. This gradual improvement in the efficiency or complexity of behaviour is referred to as cumulative culture [116], conceived to operate via a ‘ratchet effect’ [117] where beneficial variants are retained in the population until even more beneficial variants arise. In our schematic in figure 3, all of our proposed pathways can lead to such increasingly better navigational solutions over repeated rounds of innovation, retention and transmission. The fact that many wrongs and emergent sensing are able to generate information that no individual may be capable of generating on its own (i.e. these mechanisms rely on collective intelligence) suggests that they may create either overall more effective culturally transmitted traits or may generate them faster than the pathway through individual innovation, leadership and social learning. Nonetheless, both pathways suggest an important role for turnover in group membership in providing the ‘noise’ necessary for increasingly superior navigational solutions to emerge over time.

Cumulative culture is frequently claimed to be a human-unique trait [118,119], absent from other species through necessitating a suite of sophisticated socio-cognitive functions the combination of which only humans are argued to possess. To tackle the validity of this assumption, Sasaki & Biro [94] replicated a design previously used to study cumulative culture in humans experimentally [120], but with navigating pigeon flocks. The researchers removed and replaced birds in co-navigating pairs in stages, all tasked with finding a homing route from a specific release site, and found that flocks gradually improved their navigational performance across ‘generations’, reaching greater efficiencies than any control individual was capable of reaching on its own. In other words, knowledge about increasingly better travel routes appeared to accumulate through collective learning, and be passed on horizontally between individuals in groups and also vertically across generations through social learning.

Thus, we find signatures not only of culture, but also of cumulative culture in the development and maintenance of animal travel routes. Nonetheless, many open questions remain as to the true scope of such examples, both taxonomically and in terms of interactions with ecological and genetic effects. If present, cultural processes can have far-reaching consequences on a species' ecology and evolution. For example, when cultural differences between groups include the emergence of distinct migratory travel routes and strong migratory connectivity between breeding and overwintering grounds [36], they may play a role in driving and maintaining reproductive isolation between sub-populations [89], potentially affecting the evolution of the species.

Can we make predictions regarding in which species, contexts or on what scales we might expect to find migratory cultures? We suggest that a number of factors may promote the phenomenon. The ability to learn (either socially or collectively) in the context of collective movement is an essential prerequisite, as is a social structure that promotes the repeated mixing of less and more informed individuals (e.g. overlapping generations). The need to navigate to and from targets that are relatively persistent over time (e.g. to long-distance migratory destinations rather than to ephemeral food patches), but which can be reached by multiple selectively neutral alternative paths, is also likely to facilitate the emergence of stable, socially transmitted travel routes. As local cultural innovations—points of origin for inter-group variation—can arise either from individual invention or from collective intelligence, every pathway we illustrate in figure 3 has the potential to support cultural evolution. For migratory cultures to become cumulative, we suggest that what is important is the capacity to transmit routes with sufficiently high fidelity to enable beneficial modifications to accumulate gradually, in a ‘ratchet’-like fashion [117]. Such high-fidelity transmission may require (i) individual cognitive capacities to memorize landscape or other navigational cues in sufficient detail to recapitulate previously travelled routes, (ii) environments that provide such cues at sufficient resolution and (iii) terrains that permit some degree of open-endedness in route structure.

## 6. Outlook and future directions

Now is an exciting time to study collective navigation. Although in this review, we have emphasized empirical results, currently the theoretical predictions of collective navigation far outweigh empirical demonstrations. However, this asymmetry is already being eroded by emerging technologies, such as micro GPS tags, acoustic cameras, computer vision, UAVs and remote sensing satellites [121]. These technologies allow for the quantification of animal movement at extremely fine spatial and temporal scales, and in many cases it is possible to simultaneously capture the trajectories of every animal in a group in the wild. Additionally, new technologies enable us to quantify to an astonishingly fine scale the physical environments in which these animals are moving (for example, of the order of approx. 1 cm [122]). Complementing these new technologies, analytical techniques have been developed to use the data to infer the nature of social interactions [7] and leadership structures [123] within groups, and also to explore the simultaneous effects of environmental and social drivers of collective movement [6,113,124,125]. In the context of collective navigation, many open questions remain (box 2) and we are poised to make landmark discoveries—principally in understanding how animals combine social and environmental cues to find their way when navigating *through their natural habitat*.

Box 2. **Open questions for future research.**

*Do collective navigational mechanisms correlate with navigational cues or life histories?* To what extent, and how, do navigational cues (e.g. magnetic field versus landmarks) and life histories (e.g. semelparity versus iteroparity) determine which collective navigation mechanisms animals use?
*What are the mechanisms underlying distributed sensing in the wild?* UAVs and other new technologies allow us to fine-scale trajectories of many group members simultaneously [7,121] and at the same time quantify the environment in which those animals are moving in fine detail [113,122]. Combining these technologies will allow us to explore how animals combine environmental and social information when navigating in the wild.
*Do migratory insects benefit from collective navigation?* There are numerous migratory insects [126], and many of these travel at high densities and thus may benefit from collective navigation [127]. Further, they might benefit from collective navigation even when not at high densities [29]. With the possible exception of locusts, the role of social interactions in long-distance insect navigation is not well understood.
*Do animals benefit from collective decision-making to optimally time their migrations?* Correctly timing a migration is vital for survival in many species (e.g. [128]). Just as each individual may have an independent estimate of what direction to take, each individual might have an independent assessment of *when* to go. Social interactions do influence the timing of migration behaviour [129,130]. Time is distinct from space in that it is one-dimensional and asymmetric, yet many of the mechanisms for spatial collective navigation (box 1) may have temporal analogues that could help social migrants optimally time their migrations.
*How do collectively moving individuals sort into destination-specific groups?* To benefit from collective navigation, presumably individuals must have the same preferred target as the other individuals in the group, yet many fission–fusion populations mix, for example, on their wintering grounds. How do animals know when to average disparate headings and when to split up? When they do split up, how do animals effectively sort into destination-specific groups?
*What are the population genetic signatures of collective navigation?* Collective navigation during breeding migrations is predicted to lead to density-dependent dispersal [17]. An exciting possibility is that the resulting density-dependent dispersal may leave a population-genetic signature, which has yet to be quantified, but that could help identify the importance of social processes during navigation from genetic data alone.
*What is the relationship between population density and group size?* The positive feedbacks between declining population size and reduced collective navigation stem from an assumption that as populations decline so will group sizes. However, it is unknown whether as population size decreases there are fewer groups (of the same size) or a similar number of smaller groups.
*What are the population- and ecological-level consequences of collective navigation?* Theory suggests that migratory populations reliant on collective navigation may be prone to sudden population collapse and hysteresis [29,50,99]. Empirical tests of these predictions (e.g. [100]) could yield important insights for conservation and management.
*How will collective navigation shape adaptation (or not) to the Anthropocene?* How will collectively navigating species fare in a world that is increasingly affected by human activities, including temperature shifts, pollution and reduction and fragmentation of habitat? Will pollutants masking natural odours make collective navigation more important? Do pollutants have the potential to alter social behaviour enough to disrupt collective navigation [131]? Will human development lead to ‘navigational traps’ [132]? Will collective navigation help or hinder species to adapt to changes in the optimal timing and location of migrations [133]?
*Is there cumulative migratory culture in non-human animals?* We see evidence of animal migratory culture [87,91,96] and experiments suggest that it can even exhibit cumulative improvement in efficiency over time [94], but can we find evidence for such cumulative navigational culture in natural populations? Furthermore, in line with widely used definitions of cumulative culture (e.g. [119]), do we also see evidence of increases in the *complexity* of the knowledge that is transmitted? Could, for example, collective memory allow migrating populations to incorporate a greater number of landmarks into a learnt route than what any one individual could memorize?

Many group-moving taxa are under-explored in terms of collective navigation. Moreover, for taxa that have been investigated the data have often been indirect or in an artificial setting. The emerging technologies described above should allow for direct exploration of the mechanism(s) underlying collective navigation in a wide range of taxa including cetaceans, marine fishes, bats and ungulates all of which migrate and forage in large groups. Beyond increasing our understanding of their life histories, this may reveal additional mechanisms leading to group-level navigation and search. Another nearly completely unexplored taxon in terms of collective navigation is invertebrates, with the exception of the eusocial insects. Butterflies [134,135], dragonflies [136], locusts [137] and lobsters [138], among others, travel in large groups or at high densities [126,127]; however, to our knowledge if, and how, they might benefit from collective navigation have not been addressed. Migratory insects may benefit from many wrongs when selecting a migratory direction, from improved collective decision-making when deciding when weather conditions (e.g. wind direction) are favourable for efficient travel, or from emergent sensing when selecting the altitude with optimal winds. We hypothesize that context-dependent social behaviour may also contribute to desert locusts' and mormon crickets' ability to navigate out of nutritionally poor areas. These insects, which are normally herbivorous, turn to cannibalism when local vegetation is severely depleted [139]. This switch to cannibalism dramatically alters social interactions [140]. The allure of a nutritious abdomen in front and the threat of being bitten from behind tend to polarize these swarms into a forced march [141]. Individual locusts exhibit diffusive movement, which has displacement that scales as the square root of time. By contrast, the polarized groups travel in straighter paths [142]—i.e. ballistic movement, which has linear displacement. Thus even if incidental, this emergent collective effect could function to move locust populations out of barren areas more rapidly, and provide another fitness benefit for cannibalism [137].

As animals travel, even during goal-oriented movement such as long-distance migrations, navigational accuracy will not be the only selective pressure they face. In addition to navigation, animals in nature often must simultaneously balance multiple tasks while migrating, including foraging, predator avoidance and optimal energy allocation. Animals are effectively moving through complex topographies of risk, foraging opportunities, energy expenditure and physical terrain, and so their optimal movement will reflect some balance of all of these constraints along with their eventual intended target. Thus, the assumption that the shortest path between two points is the most beneficial may be incorrect. The ultimate goal of researchers should be to integrate navigation with natural history, ecology, aero-/hydrodynamics and geography when linking fine-scale (collective) movement decisions to long-range travel [71,143].

An outstanding challenge is to link a mechanistic understanding of collective navigation to population- and ecological-level processes. Explicitly considering collective effects may dramatically change predictions of models currently used to inform management and conservation [144]. For example, sudden population collapse and hysteresis are predicted by (phenomenological) models in which migration success is dependent on social learning [50,51], leadership [29] and many wrongs or emergent sensing [99]. Such predictions are consistent with empirical data suggesting that population size and migratory status are linked [145] and population collapse is associated with group travel in birds and fishes [100]. On the other hand, collective navigation could lead to density-dependent dispersal [17], and models predict that this density dependence should increase the robustness of metapopulations [146]. Collective navigation may also strongly affect genetic mixing within a population, by modulating the degree of migratory connectivity between breeding grounds and overwintering grounds [147–150], or the degree of partial migration [151,152]. In the context of a changing climate, the cultural transmission of migration routes and destinations across generations can contribute to conservative and inflexible behaviour, minimizing the ability to bet-hedge in an increasingly unpredictable climate, although the social learning of adaptive innovations within a generation can also yield a greater ability to adapt to change [133].

The study of collective behaviour typically focuses on its benefits, but there may be cases where it is maladaptive. Good decision-making in one context may be poor in another. Specifically, if a collective navigational strategy evolved to match a specific environment, anthropogenic modifications to that environment could disrupt the benefits of collective navigation and even make it a harmful strategy in the modern world. Indeed, Sigaud *et al.* [132] revealed that, in a plains bison (*Bison bison bison*) population, information transfer mediated by fission–fusion dynamics—which presumably historically transmitted beneficial information about foraging areas—in contemporary times accelerated that population's use of an ecological trap, triggering a precipitous population decline. Along those same lines, Lemasson *et al.* [153] showed that schooling may impede the downstream passage of juvenile anadromous fish through artificial barriers, increasing the time they spend in this highly risky novel habitat.

Collective navigation applies not only to large-scale orientational tasks such as migrations but also to a wide range of other behavioural contexts. Navigation is important for locating new sources of food, seeking new shelters or any other task where animals must use noisy environmental information to make decisions about where to go. Additionally, although the mechanisms may be different, there are probably rich parallels between collective search in animals and collective sensing in single-celled organisms and even groups of cells within an organism [154]. Finally, all of these biological systems may yield mechanisms, ‘discovered’ by eons of evolution, that could provide lessons and inspiration for human technologies [155], such as swarm robotics and particle swarm optimization.

## Data accessibility

This article has no additional data.

## References

[1] Altizer S, Bartel R, Han BA 2011 Animal migration and infectious disease risk. Science 331, 296–302. (10.1126/science.1194694) 21252339

[2] Bauer S, Hoye BJ 2014 Migratory animals couple biodiversity and ecosystem functioning worldwide. Science 344, 1242552 (10.1126/science.1242552) 24700862

[3] Dingle H, Drake VA 2007 What is migration? Bioscience 57, 113–121. (10.1641/B570206)

[4] Gould JL, Gould CG 2012 Nature's compass: the mystery of animal navigation. Princeton, NJ: Princeton University Press.

[5] Milner-Gulland E, Fryxell JM, Sinclair AR 2011 Animal migration: a synthesis. Oxford, UK: Oxford University Press.

[6] Dalziel BD, Corre ML, Côté SD, Ellner SP 2016 Detecting collective behaviour in animal relocation data, with application to migrating caribou. Methods Ecol. Evol. 7, 30–41. (10.1111/2041-210X.12437)

[7] Torney CJ, Lamont M, Debell L, Angohiatok RJ, Leclerc L-M, Berdahl AM 2018 Inferring the rules of social interaction in migrating caribou. Phil. Trans. R. Soc. B 373, 20170385 (10.1098/rstb.2017.0385) 29581404 PMC5882989

[8] Simons AM 2004 Many wrongs: the advantage of group navigation. Trends. Ecol. Evol. (Amst.) 19, 453–455. (10.1016/j.tree.2004.07.001) 16701304

[9] Conradt L, Roper TJ 2005 Consensus decision making in animals. Trends. Ecol. Evol. (Amst.) 20, 449–456. (10.1016/j.tree.2005.05.008) 16701416

[10] Galef BG, Laland KN 2005 Social learning in animals: empirical studies and theoretical models. AIBS Bull. 55, 489–499.

[11] Condorcet Md 1976 Essay on the application of mathematics to the theory of decision-making. In *Condorcet: Selected Writings* (ed. Baker KMl). Reprinted Indianapolis, IN, USA: Bobbs Merrill.

[12] Galton F 1907 Vox populi (The wisdom of crowds). Nature 75, 450–451. (10.1038/075450a0)

[13] Bergman G, Donner KO 1964 An analysis of the spring migration of the common scoter and the long-tailed duck in southern Finland. Acta Zool. Fennica 105, 1–59.

[14] Hamilton WJ. 1967 Social aspects of bird orientation mechanisms. In Animal Orientation and Navigation (ed. Storm RM), pp. 57–59. Corvallis, OR, USA: Oregon State University Press.

[15] Wallraff HG 1978 Social interrelations involved in migratory orientation of birds: possible contribution of field studies. Oikos 30, 401–404. (10.2307/3543490)

[16] Larkin PA, Walton A 1969 Fish school size and migration. J. Fisheries Board of Canada 26, 1372–1374. (10.1139/f69-121)

[17] Berdahl A, Westley PA, Levin SA, Couzin ID, Quinn TP 2016 A collective navigation hypothesis for homeward migration in anadromous salmonids. Fish Fisheries 17, 525–542. (10.1111/faf.12084)

[18] Seeley TD, Buhrman SC 1999 Group decision making in swarms of honey bees. Behav. Ecol. Sociobiol. (Print) 45, 19–31. (10.1007/s002650050536)

[19] Kao AB, Couzin ID 2014 Decision accuracy in complex environments is often maximized by small group sizes. Proc. R. Soc. B 281, 20133305 (10.1098/rspb.2013.3305) PMC4043084 24759858

[20] Aoki I 1982 A simulation study on the schooling mechanism in fish. Bull. Jpn. Soc. Sci. Fish. 48, 1081–1088. (10.2331/suisan.48.1081)

[21] Reynolds CW 1987 Flocks, herds and schools: a distributed behavioral model. ACM SIGGRAPH Comput. Graphics 21, 25–34. (10.1145/37402.37406)

[22] Vicsek T, Czirók A, Ben-Jacob E, Cohen I, Shochet O 1995 Novel type of phase transition in a system of self-driven particles. Phys. Rev. Lett. 75, 1226 (10.1103/PhysRevLett.75.1226) 10060237

[23] Couzin ID, Krause J, James R, Ruxton GD, Franks NR 2002 Collective memory and spatial sorting in animal groups. J. Theor. Biol. 218, 1–11. (10.1006/jtbi.2002.3065) 12297066

[24] Couzin ID, Krause J, Franks NR, Levin SA 2005 Effective leadership and decision-making in animal groups on the move. Nature 433, 513–516. (10.1038/nature03236) 15690039

[25] Hein AM, Rosenthal SB, Hagstrom GI, Berdahl A, Torney CJ, Couzin ID 2015 The evolution of distributed sensing and collective computation in animal populations. eLife 4, e10955 (10.7554/eLife.10955) 26652003 PMC4755780

[26] Ballerini M *et al.* 2008 Interaction ruling animal collective behavior depends on topological rather than metric distance: Evidence from a field study. Proc. Natl Acad. Sci. USA 105, 1232–1237. (10.1073/pnas.0711437105) 18227508 PMC2234121

[27] Strandburg-Peshkin A *et al.* 2013 Visual sensory networks and effective information transfer in animal groups. Curr. Biol. 23, R709–R711. (10.1016/j.cub.2013.07.059) 24028946 PMC4780851

[28] Rosenthal SB, Twomey CR, Hartnett AT, Wu HS, Couzin ID 2015 Revealing the hidden networks of interaction in mobile animal groups allows prediction of complex behavioral contagion. Proc. Natl Acad. Sci. USA 112, 4690–4695. (10.1073/pnas.1420068112) 25825752 PMC4403201

[29] Guttal V, Couzin ID 2010 Social interactions, information use, and the evolution of collective migration. Proc. Natl Acad. Sci. USA 107, 16 172–16 177. (10.1073/pnas.1006874107) 20713700 PMC2941337

[30] Grünbaum D 1998 Schooling as a strategy for taxis in a noisy environment. Evol. Ecol. 12, 503–522. (10.1023/A:1006574607845)

[31] Codling E, Pitchford J, Simpson S 2007 Group navigation and the ‘many-wrongs principle’ in models of animal movement. Ecology 88, 1864–1870. (10.1890/06-0854.1) 17645033

[32] Codling EA, Bode NW 2014 Copycat dynamics in leaderless animal group navigation. Mov. Ecol. 2, 11 (10.1186/2051-3933-2-11)

[33] Codling EA, Bode NW 2016 Balancing direct and indirect sources of navigational information in a leaderless model of collective animal movement. J. Theor. Biol. 394, 32–42. (10.1016/j.jtbi.2016.01.008) 26801875

[34] Bode NW, Wood AJ, Franks DW 2012 Social networks improve leaderless group navigation by facilitating long-distance communication. Curr. Zool. 58, 329–341. (10.1093/czoolo/58.2.329)

[35] Flack A, Biro D, Guilford T, Freeman R 2015 Modelling group navigation: transitive social structures improve navigational performance. J. R. Soc. Interface 12, 20150213 (10.1098/rsif.2015.0213) 26063820 PMC4528586

[36] Webster MS, Marra PP, Haig SM, Bensch S, Holmes RT 2002 Links between worlds: unraveling migratory connectivity. Trends. Ecol. Evol. (Amst.) 17, 76–83. (10.1016/S0169-5347(01)02380-1)

[37] Biro D, Sumpter DJ, Meade J, Guilford T 2006 From compromise to leadership in pigeon homing. Curr. Biol. 16, 2123–2128. (10.1016/j.cub.2006.08.087) 17084696

[38] Strandburg-Peshkin A, Farine DR, Couzin ID, Crofoot MC 2015 Shared decision-making drives collective movement in wild baboons. Science 348, 1358–1361. (10.1126/science.aaa5099) 26089514 PMC4801504

[39] Conradt L, Krause J, Couzin ID, Roper TJ 2009 ‘Leading according to need’ in self-organizing groups. Am. Nat. 173, 304–312. (10.1086/596532) 19199520

[40] Freeman R, Mann R, Guilford T, Biro D 2011 Group decisions and individual differences: route fidelity predicts flight leadership in homing pigeons (Columba livia). Biol. Lett. 7, 63–66. (10.1098/rsbl.2010.0627) 20810431 PMC3030898

[41] Romey WL 1996 Individual differences make a difference in the trajectories of simulated schools of fish. Ecol. Modell. 92, 65–77. (10.1016/0304-3800(95)00202-2)

[42] Huse G, Railsback S, Feronö A 2002 Modelling changes in migration pattern of herring: collective behaviour and numerical domination. J. Fish. Biol. 60, 571–582. (10.1006/jfbi.2002)

[43] Janson S, Middendorf M, Beekman M 2005 Honeybee swarms: how do scouts guide a swarm of uninformed bees? Anim. Behav. 70, 349–358. (10.1016/j.anbehav.2004.10.018)

[44] Couzin ID, Ioannou CC, Demirel G, Gross T, Torney CJ, Hartnett A, Conradt L, Levin SA, Leonard NE 2011 Uninformed individuals promote democratic consensus in animal groups. Science 334, 1578–1580. (10.1126/science.1210280) 22174256

[45] Hartnett AT, Schertzer E, Levin SA, Couzin ID 2016 Heterogeneous preference and local nonlinearity in consensus decision making. Phys. Rev. Lett. 116, 038701 (10.1103/PhysRevLett.116.038701) 26849620

[46] Torney CJ, Levin SA, Couzin ID 2010 Specialization and evolutionary branching within migratory populations. Proc. Natl Acad. Sci. USA 107, 20 394–20 399. (10.1073/pnas.1014316107) PMC2996695 21059935

[47] Rendell L, Fogarty L, Hoppitt WJ, Morgan TJ, Webster MM, Laland KN 2011 Cognitive culture: theoretical and empirical insights into social learning strategies. Trends. Cogn. Sci. (Regul. ed.) 15, 68–76. (10.1016/j.tics.2010.12.002) 21215677

[48] Hamilton WJ 1962 Evidence concerning the function of nocturnal call notes of migratory birds. Condor. 64, 390–401.

[49] Merkle JA, Sigaud M, Fortin D. 2015 To follow or not? How animals in fusion–fission societies handle conflicting information during group decision-making. Ecology letters 18, 799–806. 26013202 10.1111/ele.12457

[50] Fagan WF, Cantrell RS, Cosner C, Mueller T, Noble AE 2012 Leadership, social learning, and the maintenance (or collapse) of migratory populations. Theor. Ecol. 5, 253–264. (10.1007/s12080-011-0124-2)

[51] De Luca G, Mariani P, MacKenzie BR, Marsili M 2014 Fishing out collective memory of migratory schools. J. R. Soc. Interface 11, 20140043 (10.1098/rsif.2014.0043) 24647905 PMC4006244

[52] Kao AB, Miller N, Torney C, Hartnett A, Couzin ID 2014 Collective learning and optimal consensus decisions in social animal groups. PLoS. Comput. Biol. 10, e1003762 (10.1371/journal.pcbi.1003762) 25101642 PMC4125046

[53] Biro D, Sasaki T, Portugal SJ 2016 Bringing a time–depth perspective to collective animal behaviour. Trends. Ecol. Evol. (Amst.) 31, 550–562. (10.1016/j.tree.2016.03.018) 27105543

[54] Chapman JW, Nesbit RL, Burgin LE, Reynolds DR, Smith AD, Middleton DR, Hill JK 2010 Flight orientation behaviors promote optimal migration trajectories in high-flying insects. Science 327, 682–685. (10.1126/science.1182990) 20133570

[55] Couzin ID 2007 Collective minds. Nature 445, 715–715. (10.1038/445715a) 17301775

[56] Torney C, Neufeld Z, Couzin ID 2009 Context-dependent interaction leads to emergent search behavior in social aggregates. Proc. Natl Acad. Sci. USA 106, 22 055–22 060. (10.1073/pnas.0907929106) PMC2799714 20018696

[57] Torney CJ, Berdahl A, Couzin ID 2011 Signalling and the evolution of cooperative foraging in dynamic environments. PLoS Comput. Biol. 7, e1002194 (10.1371/journal.pcbi.1002194) 21966265 PMC3178622

[58] Berdahl A, Torney CJ, Ioannou CC, Faria JJ, Couzin ID 2013 Emergent sensing of complex environments by mobile animal groups. Science 339, 574–576. (10.1126/science.1225883) 23372013

[59] Keeton WT 1970 Comparative orientational and homing performances of single pigeons and small flocks. Auk 87, 797–799.

[60] Liechti F, Ehrich D, Bruderer B 1996 Flight behaviour of white storks Ciconia ciconia on their migration over southern Israël. ARDEA-WAGENINGEN- 84, 3–14.

[61] Benvenuti S, Wallraff HG 1985 Pigeon navigation: site simulation by means of atmospheric odours. J. Comp. Physiol. A: Neuroethology Sensory Neural Behav. Physiol. 156, 737–746. (10.1007/BF00610827)

[62] Rabøl J, Noer H 1973 Spring migration in the skylark (*Alauda arvensis*) in Denmark: influence of environmental factors on the flocksize and correlation between flocksize and migratory direction. Vogelwarte 27, 50–65.

[63] Tamm S 1980 Bird orientation: single homing pigeons compared with small flocks. Behav. Ecol. Sociobiol. (Print) 7, 319–322. (10.1007/BF00300672)

[64] Dell'Ariccia G, Dell'Omo G, Wolfer DP, Lipp HP 2008 Flock flying improves pigeons' homing: GPS track analysis of individual flyers versus small groups. Anim. Behav. 76, 1165–1172. (10.1016/j.anbehav.2008.05.022)

[65] Nesterova AP, Flack A, van Loon EE, Marescot Y, Bonadonna F, Biro D 2014 Resolution of navigational conflict in king penguin chicks. Anim. Behav. 93, 221–228. (10.1016/j.anbehav.2014.04.031)

[66] Irisson JO, Paris CB, Leis JM, Yerman MN 2015 With a little help from my friends: group orientation by larvae of a Coral Reef Fish. PLoS ONE 10, e0144060 (10.1371/journal.pone.0144060) 26625164 PMC4666641

[67] Ward AJ, Herbert-Read JE, Sumpter DJ, Krause J 2011 Fast and accurate decisions through collective vigilance in fish shoals. Proc. Natl Acad. Sci. USA 108, 2312–2315. (10.1073/pnas.1007102108) 21262802 PMC3038776

[68] Faria JJ, Codling EA, Dyer JR, Trillmich F, Krause J. Navigation in human crowds; testing the many-wrongs principle. Animal Behaviour 78, 587–591.

[69] Mueller T, O'Hara RB, Converse SJ, Urbanek RP, Fagan WF 2013 Social learning of migratory performance. Science 341, 999–1002. (10.1126/science.1237139) 23990559

[70] Webster MM, Whalen A, Laland KN 2017 Fish pool their experience to solve problems collectively. Nat. Ecol. Evol. 1, 0135 (10.1038/s41559-017-0135) 28812697

[71] Nagy M, Couzin ID, Fiedler W, Wikelski M, Flack A. 2018 Synchronization, coordination and collective sensing during thermalling flight of freely migrating white storks. Phil. Trans. R. Soc. B 373, 20170011 (10.1098/rstb.2017.0011) 29581396 PMC5882981

[72] Huse G, Fernö A, Holst JC 2010 Establishment of new wintering areas in herring co-occurs with peaks in the ‘first time/repeat spawner’ ratio. Mar. Ecol. Prog. Ser. 409, 189–189. (10.3354/meps08620)

[73] Flack A, Pettit B, Freeman R, Guilford T, Biro D 2012 What are leaders made of? The role of individual experience in determining leader–follower relations in homing pigeons. Anim. Behav. 83, 703–709. (10.1016/j.anbehav.2011.12.018)

[74] Macdonald J, Logemann K, Krainski ET, Sigurðsson Þ, Beale CM, Huse G, Hjøllo SS, Marteinsdóttir G 2017 Can collective memories shape fish distributions? A test, linking space-time occurrence models and population demographics. Ecography (10.1111/ecog.03098)

[75] Chernetsov N, Berthold P, Querner U 2004 Migratory orientation of first-year white storks (*Ciconia ciconia*): inherited information and social interactions. J. Exp. Biol. 207, 937–943. (10.1242/jeb.00853) 14766952

[76] Mellone U, Lucia G, Mallìa E, Urios V 2016 Individual variation in orientation promotes a 3000-km latitudinal change in wintering grounds in a long-distance migratory raptor. Ibis 158, 887–893. (10.1111/ibi.12401)

[77] Schüz E 1949 Die Spät-Auflassung ostpreussischer Jungstörche in Westdeutschland 1933. Vogelwarte 15, 63–78.

[78] Brent LJ, Franks DW, Foster EA, Balcomb KC, Cant MA, Croft DP 2015 Ecological knowledge, leadership, and the evolution of menopause in killer whales. Curr. Biol. 25, 746–750. (10.1016/j.cub.2015.01.037) 25754636

[79] Reebs SG 2000 Can a minority of informed leaders determine the foraging movements of a fish shoal? Anim. Behav. 59, 403–409. (10.1006/anbe.1999.1314) 10675263

[80] Lewis JS, Wartzok D, Heithaus MR 2011 Highly dynamic fission–fusion species can exhibit leadership when traveling. Behav. Ecol. Sociobiol. (Print) 65, 1061–1069. (10.1007/s00265-010-1113-y)

[81] Payne K 2003 Sources of social complexity in the three elephant species. Cambridge, MA: Harvard University Press.

[82] Miller N, Garnier S, Hartnett AT, Couzin ID 2013 Both information and social cohesion determine collective decisions in animal groups. Proc. Natl Acad. Sci. USA 110, 5263–5268. (10.1073/pnas.1217513110) 23440218 PMC3612658

[83] Laland KN, Williams K 1997 Shoaling generates social learning of foraging information in guppies. Anim. Behav. 53, 1161–1169. (10.1006/anbe.1996.0318) 9236013

[84] Schultz KM, Passino KM, Seeley TD 2008 The mechanism of flight guidance in honeybee swarms: subtle guides or streaker bees? J. Exp. Biol. 211, 3287–3295. (10.1242/jeb.018994) 18840663

[85] Holdo RM, Holt RD, Fryxell JM 2009 Opposing rainfall and plant nutritional gradients best explain the wildebeest migration in the Serengeti. Am. Nat. 173, 431–445. (10.1086/597229) 19243258

[86] Keefer ML, Caudill CC, Peery CA, Moser ML 2013 Context-dependent diel behavior of upstream-migrating anadromous fishes. Environ. Biol. Fishes. 96, 691–700. (10.1007/s10641-012-0059-5)

[87] Corten A 2001 The role of ‘conservatism’ in herring migrations. Rev. Fish. Biol. Fish. 11, 339–361. (10.1023/A:1021347630813)

[88] Schüz E. 1951 Überblick über die Orientierungsversuche der Vogelwarte Rossitten (jetzt: Vogelwarte Radolfzell). Proc. Xth Int. Ornithol. Congr., Uppsala, Sweden, pp. 249–268.

[89] Harrison XA *et al.* 2010 Cultural inheritance drives site fidelity and migratory connectivity in a long-distance migrant. Mol. Ecol. 19, 5484–5496. (10.1111/j.1365-294X.2010.04852.x) 21083633

[90] Perdeck A 1958 Two types of orientation in migrating starlings, *Sturnus yulgaris* L., and *Chaffinches, Fringilla coelebs* L., as Revealed by Displacement Experiments. Ardea 46, 1–2. (10.5253/arde.v1i2.p1)

[91] Helfman GS, Schultz ET 1984 Social transmission of behavioural traditions in a coral reef fish. Anim. Behav. 32, 379–384. (10.1016/S0003-3472(84)80272-9)

[92] Riley JR, Greggers U, Smith AD, Reynolds DR, Menzel R 2005 The flight paths of honeybees recruited by the waggle dance. Nature 435, 205–207. (10.1038/nature03526) 15889092

[93] Franks NR, Richardson T 2006 Teaching in tandem-running ants. Nature 439, 153–153. (10.1038/439153a) 16407943

[94] Sasaki T, Biro D 2017 Cumulative culture can emerge from collective intelligence in animal groups. Nature 8, 15049 (10.1038/ncomms15049) PMC5399285 28416804

[95] Pettit B, Flack A, Freeman R, Guilford T, Biro D 2013 Not just passengers: pigeons, Columba livia, can learn homing routes while flying with a more experienced conspecific. Proc. R. Soc. B 280, 20122160 (10.1098/rspb.2012.2160) PMC3574437 23135677

[96] Warner RR 1988 Traditionality of mating-site preferences in a coral reef fish. Nature 335, 719–721. (10.1038/335719a0)

[97] Peterson RO, Jacobs AK, Drummer TD, Mech LD, Smith DW 2002 Leadership behavior in relation to dominance and reproductive status in gray wolves, Canis lupus. Can. J. Zool. 80, 1405–1412. (10.1139/z02-124)

[98] McQuinn IH 1997 Metapopulations and the Atlantic herring. Rev. Fish Biol. Fish. 7, 297–329. (10.1023/A:1018491828875)

[99] Berdahl A, van Leeuwen A, Levin SA, Torney CJ 2016 Collective behavior as a driver of critical transitions in migratory populations. Mov. Ecol. 4, 18 (10.1186/s40462-016-0083-8) 27429757 PMC4946155

[100] Hardesty-Moore M *et al.* 2018 Migration in the Anthropocene: how collective navigation, environmental system and taxonomy shape the vulnerability of migratory species. Phil. Trans. R. Soc. B 373, 20170017 (10.1098/rstb.2017.0017) 29581401 PMC5882986

[101] Sinclair ARE 1995 Serengeti: dynamics of an ecosystem. Chicago, IL: University of Chicago Press.

[102] Harris G, Thirgood S, Hopcraft JGC, Cromsigt JP, Berger J 2009 Global decline in aggregated migrations of large terrestrial mammals. Endangered Species Res. 7, 55–76. (10.3354/esr00173)

[103] Dumond M, Lee DS 2013 Dolphin and union caribou herd status and trend. Arctic 66, 329–337. (10.14430/arctic4311)

[104] Ioannou CC 2017 Swarm intelligence in fish? The difficulty in demonstrating distributed and self-organised collective intelligence in (some) animal groups. Behav. Processes 141, 141–151. (10.1016/j.beproc.2016.10.005) 27737770

[105] Banks AN, Guilford T 2000 Accurate route demonstration by experienced homing pigeons does not improve subsequent homing performance in naive conspecifics. Proc. R. Soc. Lond. B 267, 2301–2306. (10.1098/rspb.2000.1283)

[106] Seeley TD, Visscher PK, Passino KM 2006 Group decision making in honey bee swarms. Am. Sci. 94, 220 (10.1511/2006.3.220)

[107] Pratt SC, Mallon EB, Sumpter DJ, Franks NR 2002 Quorum sensing, recruitment, and collective decision-making during colony emigration by the ant Leptothorax albipennis. Behav. Ecol. Sociobiol. (Print) 52, 117–127. (10.1007/s00265-002-0487-x)

[108] Bonnie KE, Earley RL 2007 Expanding the scope for social information use. Anim. Behav. 74, 171–181. (10.1016/j.anbehav.2006.12.009)

[109] Laland KN, Janik VM 2006 The animal cultures debate. Trends. Ecol. Evol. (Amst.) 21, 542–547. (10.1016/j.tree.2006.06.005) 16806574

[110] Whiten A, Mesoudi A 2008 Establishing an experimental science of culture: animal social diffusion experiments. Phil. Trans. R. Soc. B 363, 3477–3488. (10.1098/rstb.2008.0134) 18799418 PMC2607342

[111] Henrich J 2004 Demography and cultural evolution: how adaptive cultural processes can produce maladaptive losses—the Tasmanian case. Am. Antiq. 69, 197–214.

[112] Flack A, Biro D 2013 Collective learning in route navigation. Commun. Integr. Biol. 6, e26521 (10.4161/cib.26521) 24505504 PMC3913685

[113] Strandburg-Peshkin A, Farine DR, Crofoot MC, Couzin ID 2017 Habitat and social factors shape individual decisions and emergent group structure during baboon collective movement. eLife 6, e19505 (10.7554/eLife.19505.001) 28139196 PMC5283833

[114] King JG, Cooper BA, Ritchie RJ 1998 Mixed-species Swan flocks migrating in East-Central Alaska. Northwestern Nat. 79, 104–107. (10.2307/3536839)

[115] Sridhar H, Guttal V 2018 Friendship across species borders: factors that facilitate and constrain heterospecific sociality. Phil. Trans. R. Soc. B 373, 20170014 (10.1098/rstb.2017.0014) 29581399 PMC5882984

[116] Boyd R, Richerson P. 1996 Why culture is common, but cultural evolution is rare. Proceedings of the British Academy 88, 77–93.

[117] Tomasello M, Kruger AC, Ratner HH 1993 Cultural learning. Behav. Brain Sci. 16, 495–511. (10.1017/S0140525X0003123X)

[118] Tennie C, Call J, Tomasello M 2009 Ratcheting up the ratchet: on the evolution of cumulative culture. Phil. Trans. R. Soc. B 364, 2405–2415. (10.1098/rstb.2009.0052) 19620111 PMC2865079

[119] Dean LG, Vale GL, Laland KN, Flynn E, Kendal RL 2014 Human cumulative culture: a comparative perspective. Biol. Rev. 89, 284–301. (10.1111/brv.12053) 24033987

[120] Caldwell CA, Millen AE 2008 Experimental models for testing hypotheses about cumulative cultural evolution. Evol. Hum. Behav. 29, 165–171. (10.1016/j.evolhumbehav.2007.12.001)

[121] Hughey LF, Hein AM, Strandburg-Peshkin A, Jensen FH 2018 Challenges and solutions for studying collective animal behaviour in the wild. Phil. Trans. R. Soc. B 373, 20170005 (10.1098/rstb.2017.0005) 29581390 PMC5882975

[122] Fraser RH, Olthof I, Lantz TC, Schmitt C 2016 UAV photogrammetry for mapping vegetation in the low-Arctic. Arct. Sci. 2, 79–102. (10.1139/as-2016-0008)

[123] Strandburg-Peshkin A, Papageorgiou D, Crofoot MC, Farine DR 2018 Inferring influence and leadership in moving animal groups. Phil. Trans. R. Soc. B 373, 20170006 (10.1098/rstb.2017.0006) 29581391 PMC5882976

[124] Bode NW, Franks DW, Wood AJ, Piercy JJ, Croft DP, Codling EA 2012 Distinguishing social from nonsocial navigation in moving animal groups. Am. Nat. 179, 621–632. (10.1086/665005) 22504544

[125] Calabrese JM, Fleming CH, Fagan WF, Rimmler M, Kaczensky P, Bewick S, Leimgruber P, Mueller T 2018 Disentangling social interactions and environmental drivers in multi-individual wildlife tracking data. Phil. Trans. R. Soc. B 373, 20170007 (10.1098/rstb.2017.0007) 29581392 PMC5882977

[126] Holland RA, Wikelski M, Wilcove DS 2006 How and why do insects migrate? Science 313, 794–796. (10.1126/science.1127272) 16902129

[127] Hu G, Lim KS, Horvitz N, Clark SJ, Reynolds DR, Sapir N, Chapman JW 2016 Mass seasonal bioflows of high-flying insect migrants. Science 354, 1584–1587. (10.1126/science.aah4379) 28008067

[128] Satterthwaite WH, Carlson SM, Allen-Moran SD, Vincenzi S, Bograd SJ, Wells BK 2014 Match-mismatch dynamics and the relationship between ocean-entry timing and relative ocean recoveries of Central Valley fall run Chinook salmon. Mar. Ecol. Prog. Ser. 511, 237–248. (10.3354/meps10934)

[129] Helm B, Piersma T, Van der Jeugd H 2006 Sociable schedules: interplay between avian seasonal and social behaviour. Anim. Behav. 72, 245–262. (10.1016/j.anbehav.2005.12.007)

[130] Berdahl A, Westley PA, Quinn TP 2017 Social interactions shape the timing of spawning migrations in an anadromous fish. Anim. Behav. 126, 221–229.

[131] Brodin T, Fick J, Jonsson M, Klaminder J 2013 Dilute concentrations of a psychiatric drug alter behavior of fish from natural populations. Science 339, 814–815. (10.1126/science.1226850) 23413353

[132] Sigaud M, Merkle JA, Cherry SG, Fryxell JM, Berdahl A, Fortin D 2017 Collective decision-making promotes fitness loss in a fusion-fission society. Ecol. Lett. 20, 33–40. (10.1111/ele.12698) 27873440

[133] Keith SA, Bull JW 2017 Animal culture impacts species' capacity to realise climate-driven range shifts. Ecography 40, 296–304. (10.1111/ecog.02481)

[134] Brower LP. 1985 New perspectives on the migration biology of the monarch butterfly, *Danaus plexippus* L. In Migration: mechanisms and adaptive significance (ed. Rankin MA), pp. 748–785. Austin, TX, USA: University of Texas Contributions to Marine Science.

[135] Frey D, Leong K, Fredericks D, Raskowitz S 1992 Clustering patterns of monarch butterflies (Lepidoptera: Danaidae) at two California central coast overwintering sites. Ann. Entomol. Soc. Am. 85, 148–153. (10.1093/aesa/85.2.148)

[136] Russell RW, May ML, Soltesz KL, Fitzpatrick JW 1998 Massive swarm migrations of dragonflies (Odonata) in eastern North America. Am. Midl. Nat. 140, 325–342. (10.1674/0003-0031(1998)140%5B0325:MSMODO%5D2.0.CO;2)

[137] Hansen MJ, Buhl J, Bazazi S, Simpson SJ, Sword GA 2011 Cannibalism in the lifeboat–collective movement in Australian plague locusts. Behav. Ecol. Sociobiol. (Print) 65, 1715–1720. (10.1007/s00265-011-1179-1)

[138] Herrnkind WF, Childress MJ, Lavalli KL 2001 Cooperative defence and other benefits among exposed spiny lobsters: inferences from group size and behaviour. Mar. Freshwater Res. 52, 1113–1124. (10.1071/MF01044)

[139] Simpson SJ, Sword GA, Lorch PD, Couzin ID 2006 Cannibal crickets on a forced march for protein and salt. Proc. Natl Acad. Sci. USA 103, 4152–4156. (10.1073/pnas.0508915103) 16537500 PMC1449662

[140] Buhl J, Sumpter DJ, Couzin ID, Hale JJ, Despland E, Miller ER, Simpson SJ 2006 From disorder to order in marching locusts. Science 312, 1402–1406. (10.1126/science.1125142) 16741126

[141] Bazazi S, Buhl J, Hale JJ, Anstey ML, Sword GA, Simpson SJ, Couzin ID 2008 Collective motion and cannibalism in locust migratory bands. Curr. Biol. 18, 735–739. (10.1016/j.cub.2008.04.035) 18472424

[142] Romanczuk P, Couzin ID, Schimansky-Geier L 2009 Collective motion due to individual escape and pursuit response. Phys. Rev. Lett. 102, 010602 (10.1103/PhysRevLett.102.010602) 19257176

[143] Torney CJ, Grant C. Hopcraft J, Morrison TA, Couzin ID, Levin SA 2018 From single steps to mass migration: the problem of scale in the movement ecology of the Serengeti wildebeest. Phil. Trans. R. Soc. B 373, 20170012 (10.1098/rstb.2017.0012) 29581397 PMC5882982

[144] Westley PAH, Berdahl AM, Torney CJ, Biro D 2018 Collective movement in ecology: from emerging technologies to conservation and management. Phil. Trans. R. Soc. B 373, 20170004 (10.1098/rstb.2017.0004) PMC5882974 29581389

[145] Bolger DT, Newmark WD, Morrison TA, Doak DF 2008 The need for integrative approaches to understand and conserve migratory ungulates. Ecol. Lett. 11, 63–77. (10.1111/j.1461-0248.2007.01109.x) 17897327

[146] Yeakel JD, Gibert JP, Gross T, Westley PAH, Moore JW 2018 Eco-evolutionary dynamics, density-dependent dispersal and collective behaviour: implications for salmon metapopulation robustness. Phil. Trans. R. Soc. B 373, 20170018 (10.1098/rstb.2017.0018) 29581402 PMC5882987

[147] Bauer S, Lisovski S, Hahn S 2016 Timing is crucial for consequences of migratory connectivity. Oikos 125, 605–612. (10.1111/oik.02706)

[148] Turgeon J, Duchesne P, Colbeck GJ, Postma LD, Hammill MO 2012 Spatiotemporal segregation among summer stocks of beluga (Delphinapterus leucas) despite nuclear gene flow: implication for the endangered belugas in eastern Hudson Bay (Canada). Conserv. Genet. 13, 419–433. (10.1007/s10592-011-0294-x)

[149] BrownGladden J, Ferguson M, Clayton J 1997 Matriarchal genetic population structure of North American beluga whales Delphinapterus leucas (Cetacea: Monodontidae). Mol. Ecol. 6, 1033–1046. (10.1046/j.1365-294X.1997.00275.x) 9394462

[150] Carroll EL, Baker CS, Watson M, Alderman R, Bannister J, Gaggiotti OE, Gröcke DR, Patenaude N, Harcourt R 2015 Cultural traditions across a migratory network shape the genetic structure of southern right whales around Australia and New Zealand. Sci. Rep. 5, 16182 (10.1038/srep16182) 26548756 PMC4637828

[151] Barnowe-Meyer KK, White P, Waits LP, Byers JA 2013 Social and genetic structure associated with migration in pronghorn. Biol. Conserv. 168, 108–115. (10.1016/j.biocon.2013.09.022)

[152] Chapman BB, Brönmark C, Nilsson JÅ, Hansson LA 2011 The ecology and evolution of partial migration. Oikos 120, 1764–1775. (10.1111/j.1600-0706.2011.20131.x)

[153] Lemasson BH, Haefner JW, Bowen MD 2014 Schooling increases risk exposure for fish navigating past artificial barriers. PLoS ONE 9, e108220 (10.1371/journal.pone.0108220) 25268736 PMC4182462

[154] Haeger A, Wolf K, Zegers MM, Friedl P 2015 Collective cell migration: guidance principles and hierarchies. Trends. Cell. Biol. 25, 556–566. (10.1016/j.tcb.2015.06.003) 26137890

[155] Brambilla M, Ferrante E, Birattari M, Dorigo M 2013 Swarm robotics: a review from the swarm engineering perspective. Swarm Intell. 7, 1–41. (10.1007/s11721-012-0075-2)

